# Prevalence and Therapies of Antibiotic-Resistance in *Staphylococcus aureus*

**DOI:** 10.3389/fcimb.2020.00107

**Published:** 2020-03-17

**Authors:** Yunlei Guo, Guanghui Song, Meiling Sun, Juan Wang, Yi Wang

**Affiliations:** ^1^Department of Endocrinology, The Affiliated Hospital of Qingdao University, Qingdao, China; ^2^Department of Clinical Laboratory, The Affiliated Hospital of Qingdao University, Qingdao, China

**Keywords:** *Staphylococcus aureus*, molecular mechanisms, acquired antibiotic resistance, antibiotic resistance therapy, cell membrane

## Abstract

Infectious diseases are the second most important cause of human death worldwide; *Staphylococcus aureus (S. aureus)* is a very common human pathogenic microorganism that can trigger a variety of infectious diseases, such as skin and soft tissue infections, endocarditis, osteomyelitis, bacteremia, and lethal pneumonia. Moreover, according to the sensitivity to antibiotic drugs, *S. aureus* can be divided into methicillin-sensitive *Staphylococcus aureus* (MSSA) and methicillin-resistant *Staphylococcus aureus* (MRSA). In recent decades, due to the evolution of bacteria and the abuse of antibiotics, the drug resistance of *S. aureus* has gradually increased, the infection rate of MRSA has increased worldwide, and the clinical anti-infective treatment for MRSA has become more difficult. Accumulating evidence has demonstrated that the resistance mechanisms of *S. aureus* are very complex, especially for MRSA, which is resistant to many kinds of antibiotics. Therefore, understanding the drug resistance of MRSA in a timely manner and elucidating its drug resistance mechanism at the molecular level are of great significance for the treatment of *S. aureus* infection. A large number of researchers believe that analyzing the molecular characteristics of *S. aureus* can help provide a basis for designing effective prevention and treatment measures against hospital infections caused by *S. aureus* and further monitor the evolution of *S. aureus*. This paper reviews the research status of MSSA and MRSA, the detailed mechanisms of the intrinsic antibiotic resistance and the acquired antibiotic resistance, the advanced research on anti-MRSA antibiotics and novel therapeutic strategies for MRSA treatment.

## Introduction

### Staphylococcus Aureus

*Staphylococcus aureus (S. aureus)* is one of main pathogens in hospital and community infections and can cause many infectious diseases, such as mild skin and soft tissue infections infective endocarditis, osteomyelitis, bacteremia, and fatal pneumonia (Lowy, 1998; Humphreys, 2012). *Staphylococcus aureus* was first discovered in 1880 in Aberdeen, Scotland, by surgeon Alexander Ogston from patients with ulcerated sores. *Staphylococcus aureus* belongs to the genus Staphylococcus, Firmicutes; is positive for Gram stain, ~0.8 μm in diameter, arranged in a “string of grapes” under a microscope, an aerobic or anaerobic; and grows optimally at 37°C, and at pH7.4 (Edwards and Massey, 2011; Gardete and Tomasz, 2014). The colonies on blood agar plate are thick, shiny, and round with a diameter of 1~2 mm (Gonzalez-Perez et al., 2019; Sato et al., 2019). Most of them are hemolytic, forming a transparent hemolytic ring around the colonies on blood agar plates (Sato et al., 2019). Moreover, *S. aureus* does not form spores or flagella, but possesses a capsule, can produce golden yellow pigment, and decompose mannitol (Tayeb-Fligelman et al., 2017). Additionally, it has also been found that tests of plasma coagulase, lactose fermentation and deoxyribonuclease are positive in *S. aureus* (Chino et al., 2017; Tayeb-Fligelman et al., 2017).

### Methicillin-Resistant Staphylococcus Aureus (MRSA)

**Figure 1:**
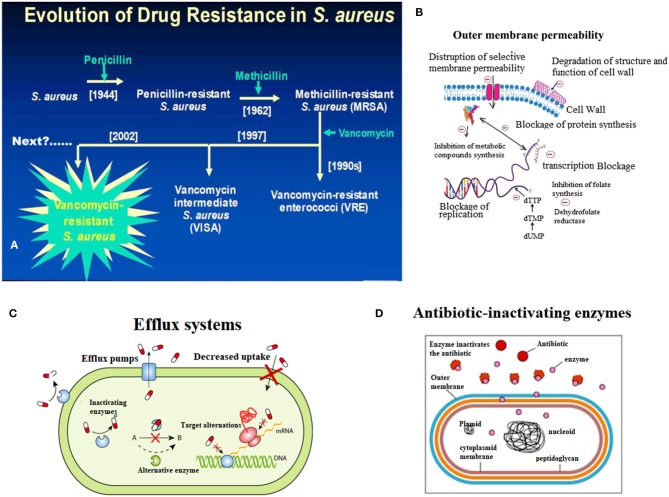
Endogenous resistance mechanism of *Staphylococcus aureus*. **(A)** Breif introduction of evolution of drug resistance in *S. aureus*. **(B)** The scheme of decreased outmembrane permeability caused drug resistance of *S. aureus*. **(C)** The role of active efflux systems in MRSA resistance. **(D)** The role of cellular enzymes in drug resistance of *S. aureus*.

Fleming discovered penicillin in the 1940s and pioneered the era of antibiotics for infection treatment (Klevens et al., 2007; Klein et al., 2017). At the time, the infectious diseases caused by *S. aureus* were well-controlled, but with the widespread use of penicillin in the 1950s, penicillin-resistant *S. aureus* appeared in the clinic (Rayner and Munckhof, 2005; Pichereau and Rose, 2010). Penicillin-resistant *S. aureus* can produce penicillinase, which can hydrolyze the penicillin β-lactam ring, leading to resistance to penicillin. Later, scientists developed a new penicillinase-resistant semisynthetic penicillin named methicillin, which is resistant to the hydrolysis of β-lactamase (Rayner and Munckhof, 2005; Khoshnood et al., 2019). After being applied to the clinic in 1959, methicillin effectively controlled the infection of penicillin-resistant *S. aureus* (Chambers and Deleo, 2009; Jokinen et al., 2017). However, only 2 years after methicillin was applied, in 1961, British scientist Jevons reported the isolation of an MRSA strain; this resistance was produced by a gene encoding the penicillin-binding protein 2a or 2′ (PBP2a or PBP2′) (mecA) which was integrated into the chromosomal element (SCCmec) of methicillin-sensitive *S. aureus* (Schulte and Munson, 2019). Moreover, MRSA has rapidly become the most frequently occurring resistant pathogen identified in many parts of the world, including Europe, the United States, North Africa, the Middle East and East Asia (Mediavilla et al., 2012; Lakhundi and Zhang, 2018). According to its original source, MRSA is classified into hospital-acquired MRSA (HA-MRSA) and community-acquired MRSA (CA-MRSA) (Lindsay, 2013; Otto, 2013). In China, the proportion of hospital-acquired MRSA has reached 50.4% (Shang et al., 2016). Additionally, based on the Centers for Disease Control (CDC) in the US, the mortality rate of MRSA infection has exceeded that of acquired immune deficiency syndrome (AIDS), Parkinson's disease and murder (Lessa et al., 2012). Thus, the analysis of the molecular characteristics of *S. aureus*, which has become the focus of global public health concerns, can help us understand the prevalence of *S. aureus*, monitor the evolution of *S. aureus*, discover new molecular features of *S. aureus*, and provide information for developing novel drugs to against *S. aureus* (Figure 1A).

## Intrinsic Antibiotic Resistance

The resistance rates of *S. aureus* infection and multidrug resistant strains are increasing, making the clinical anti-infective treatment more difficult. The endogenous resistance mechanism mainly includes three aspects (Figure 1).

### Outer Membrane Permeability

When the cell membrane permeability is lowered, the energy metabolism of the bacteria is affected, and therefore, drug absorption is reduced, leading to drug resistance (Li et al., 2013; Anuj et al., 2019). For example, the resistance of *S. aureus* to aminoglycosides is caused by a decrease in membrane permeability and finally results in a decrease in drug intake (Figure 1B) (Hori and Hiramatsu, 1994; Andrade et al., 2014).

### Efflux Systems

The active efflux system of bacteria was discovered in 1980 by Ball and McMurry when studying the resistance of *Escherichia coli* to tetracycline (Seifi and Khoshbakht, 2016). Afterwards, the scholars conducted many experiments on the active efflux system, which confirmed that the active efflux system is a normal physiological structure of bacteria, and exists in sensitive strains (Lekshmi et al., 2018). When induced by substrates in the environment for a long time, efflux system-encoding genes are activated and expressed, and the ability to efflux drugs is greatly enhanced, thus leading to drug resistance (Zarate et al., 2019). Active drug efflux systems play a role in resistance to multiple drugs (Costa et al., 2018). There are three types of multidrug-pumping proteins present on the *Staphylococcus aureus* cell membrane: QacA, NorA, and Smr (Foster, 2016; Jang, 2016). Noguchi et al. considered QacA to be an important factor in MRSA (Noguchi et al., 2005; Nakaminami et al., 2019). Multidrug pumping proteins are all proton kinesins (Foster, 2016). That is, instead of relying on ATP hydrolysis to release energy, material exchange is performed by an electrochemical gradient formed by H^+^ on both sides of the cell membrane (Matano et al., 2017). Usually, it is a reversible process, that is, H^+^ moves from extracellular to intracellular, while intracellular harmful substances such as dyes and antibacterial drugs flow from the inside of the cell to the outside (Lowy, 1998). Experiments by Kristiansen et al. also demonstrated the role of active efflux systems in MRSA resistance (Mo et al., 2007) (Figure 1C).

### Excessive Production of β-Lactamase

β-lactamase is an enzyme that catalyzes the hydrolysis of various β-lactam antibiotics (including antibiotics such as carbapenem broad-spectrum antibiotics), is encoded by bacterial chromosomal genes, and is transferable (Lee and Park, 2016). At present, the research shows that β-lactam antibiotics have a lethal effect on bacteria mainly through two mechanisms: first, by binding to penicillin-binding protein (PBPs, i.e., cell wall mucin synthase), which represses cell wall mucin synthesis, disrupts the cell wall, and leads to bacterial expansion and lysis; second, by triggering the autolytic enzyme activity of the bacteria, which resulted in autolysis and death (Matono et al., 2018). Excessive secretion of β-lactamase by MRSA mainly reduces the effect of antibiotics through two mechanisms, which lead to MRSA resistant (Khan et al., 2014). The first is the hydrolysis mechanism, that is, β-lactamase hydrolzes and inactivates β-lactam antibiotics; the second is the mechanism of pinching, that is, a large amount of β-lactamase binds quickly and firmly to extracellular antibiotics, preventing the antibiotics from reaching the intracellular space and therefore the antibiotics are not able to reach the target site, ultimately leading to MRSA resistance to antibiotics (Figure 1D) (Harada et al., 2014; Hashizume et al., 2017).

## Acquired Antibiotic Resistance

### Resistance by Mutations

*Staphylococcus aureus* can become drug-resistant by genetic mutations that alter the target DNA gyrase or reduce outer membrane proteins, thereby reducing drug accumulation (Kime et al., 2019; Yang et al., 2019). For example, the principle of resistance to clindamycin and erythromycin is caused by a modification in ribosomal RNA methylase (Heelan et al., 2004; Martinez et al., 2018).

### Acquisition of Resistance Genes

Acquired resistance is a type of plasmid-mediated resistance (Foster, 2017). Through plasmid-mediated transduction, transformation, and insertion of drug-resistant genes, excessive β-lactamase can be produced, leading to bacteria resistance (Foster, 2017; Haaber et al., 2017). The mechanism of MRSA resistance is mainly because plasmids, or drug-resistant gene transmission mediated by plasmids, which can expand the genome and resistance genes can be transferred between *S. aureus* and other bacteria (Vestergaard et al., 2019). For example, MRSA can obtain drug-resistant plasmids from *Enterococcus*, further expanding and enhancing its resistance (Lazaris et al., 2017).

### Biofilm-Mediated Resistance

Bacterial biofilm is an extracellular complex structure composed of a microbial population attached to the surface of the substrate, and its internal microorganisms are surrounded by a highly hydrated extracellular polymer matrix produced by itself, which is a protective way of survival for bacteria to adapt to their surroundings (Craft et al., 2019; Saxena et al., 2019). Moreover, the vast majority of bacteria in nature exist in the form of biofilms, and the most prominent feature of bacterial biofilms is their strong adhesion and drug resistance, which allowing bacteria to resist host immune responses and evade antibiotic killing (Kanwar et al., 2017). Their resistance to antibacterial drugs can increase to 1,000 times that of plankton. At present, domestic and foreign anti-biofilm treatments mainly focus on the continuous development of new antibacterial drugs, but antibiotics and chemical synthetic drugs used in clinical medicine have certain toxic effects (Saxena et al., 2019). Biofilm bacteria are prone to resistance to these conventional drugs, and resistant strains presented an increasing trend (Craft et al., 2019). Studies have shown that the combined application of traditional Chinese medicine and antibiotics has the advantage of reducing efficiency (Kanwar et al., 2017).

### Persister Cells in Antibiotic Resistance

Persister cells are a small subset of cells that are genetically homologous but phenotypically heterogeneous in a microbial population, grow slowly, or dormant and survive high concentrations of antibiotics (Fisher et al., 2017). Early studies suggest that, unlike antibiotic resistance, bacterial retention is a physiological state of bacteria that temporarily resists antibiotic stress, and does not result in a change in the genotype (Kester and Fortune, 2014). However, this statement is being challenged because of the rapid development of high-throughput sequencing technology. When bacteria encounter external stimuli such as antibiotics, most of the bacteria are killed immediately, but a small percentage of the bacteria will resist this pressure by arresting growth and remaining inactive (Michiels et al., 2016). When the external pressure disappears, this small amount of bacteria can return to normal growth; we call these bacteria persister cell (Fisher et al., 2017). The presence of persister cells poses many obstacles to the complete elimination of bacterial infections and the prevention of recurrent infections (Fisher et al., 2017; Foster, 2017). Bacterial persister cells exhibit antibiotic tolerance, slow growth, and the ability to restart infection after antibiotic treatment. Antibiotics can cause fatal damage to bacteria, but persister cells can resist this killing by reducing cell growth and metabolism, and even by becoming dormant (Lewis, 2008). Bacterial persistence refers to a state of reduced metabolic activity that endows a subpopulation of isogenic bacteria with multidrug tolerance (Fisher et al., 2017). Persisters are phenotypic variants but not mutants (Kester and Fortune, 2014). Existing research results showed that the mechanism of bacterial persistence is complex and the related signaling pathways include toxins-antitoxin systems, cell physiological reduction of energy metabolism and protein and nucleic acid synthesis, DNA protection and repair systems, protease systems, trans-translation, external pumping system, and so on (Michiels et al., 2016; Fisher et al., 2017).

## Research Advances in Commonly Used Antibiotics

MRSA is a kind of multidrug resistant “super bacteria” resistant to penicillins, cephalosporins, chloramphenicol, lincomycin, aminoglycosides, tetracyclines, macrolides, quinophthalones, sulfonamides and rifampicin, which is a very difficult problem in clinical treatment (2016). Moreover, it has been reported that MRSA infection is one of the world's major infectious diseases due to its high rates of morbidity and mortality, which seriously threaten human health and have attracted the attention of the global medical community (Hassoun et al., 2017). Therefore, it is urgent to find effective drugs to treat multidrug resistant bacterial infections. We have listed some of the drugs used against anti-MRSA infections, that have been applied to in the clinic in Table 1, and three of them (i.e.,: vancomycin, daptomycin, and linezolid) have been highlighted.

**Table 1:** Summaries of effects of anti-MRSA antibiotics.

**Drugs**	**Effects**	**Disadvantages**	**References**
Vancomycin	Vancomycin has long been considered the best drug for the treatment of severe MRSA infection	More adverse reactions, mainly manifested as ototoxicity, nephrotoxicity	Holmes et al., 2015
Norvancomycin	Norvancomycin is a glycopeptide antibiotic developed in China. Its pharmacological aspect is similar to vancomycin. The 0.4 g dose is equivalent to vancomycin 0.5 g, which has a good price-to-effect ratio	The effectiveness and safety of norvancomycin also requires further large-scale clinical trials	Li J. et al., 2017
Teicoplanin	Teicoplanin is another type of glycopeptide antibiotic used to treat MRSA infection. Its molecular structure, antibacterial spectrum, and antibacterial activity are similar to vancomycin. Clinically applicable to infections caused by Gram-positive bacteria resistant to penicillin and cephalosporins, or serious infections in patients allergic to β-lactam antibiotics in patients, such as bacteremia, endocarditis, skin, and soft tissue infections, lower respiratory tract infections, leukopenia, infection, etc.	Common adverse reactions are: local pain injection; nephrotoxicity similar to vancomycin, but generally mild and transient, rarely need to interrupt treatment; allergic reactions, fever, liver, and kidney dysfunction	Ramos-Martin et al., 2017
Linezolid	Linezolid has a bacteriostatic action against *Enterococcus* and *Staphylococcus*, and has a bactericidal effect on most strains of *Streptococcus*. It is mainly used to control systemic infection caused by Vancomycin-resistant *Enterococcus faecium*, including sepsis and pneumonia	Adverse reactions are mainly: digestive tract reactions, such as diarrhea, nausea, followed by headache, abnormal liver function, thrombocytopenia, hemorrhage, ulcers, fatigue, rash, vaginal candidiasis, and other fungal infections	Sazdanovic et al., 2016
Quinupristin/Dalfopristin	The antibacterial spectrum of Quinupudin/Dafupudin has good antibacterial activity against MRSA, MSSA, *Enterococcus faecium* and *Streptococcus*, and is especially suitable for infection caused by Gram-positive cocci, which is commonly used for antibiotic resistance. The antibacterial activity is comparable to or stronger than vancomycin.	Adverse reactions: local inflammatory response, pain, phlebitis, nausea, vomiting, diarrhea, joint pain, myalgia, muscle weakness, and rash	Delgado et al., 2000
Daptomycin	Daptomycin can be used to treat skin soft tissue infections and bloodstream infections caused by MRSA, but not for MRSA-induced pneumonia	Common adverse reactions were gastrointestinal reactions, injection site reactions, fever, headache, insomnia, dizziness and rash, all of which were mild to moderate	Heidary et al., 2018
Tigecycline	Tigecycline has a broad-spectrum antibacterial activity and is effective against Gram-positive or Gram-negative bacteria, especially against Gram-positive bacteria	Common adverse reactions are damage to the digestive system, such as nausea, vomiting, diarrhea, and other adverse reactions including infection, albumin reduction, and difficulty breathing	Wang et al., 2017
Ceftobiprole	It is used to treat complex skin and soft tissue infections and medical care related pneumonia. Cefepime has strong anti-MRSA and penicillin resistant *pneumococci* activities	Unclear	Horn et al., 2017
New glycopeptide (e.g., Oritavancin, Dalbavancin)	The antibacterial effect is similar to vancomycin, effective against methicillin-resistant or resistant *Staphylococcus*, penicillin-resistant *pneumococci* and *enterococci*, and oritavancin is effective against vancomycin-resistant pathogens, while dalbavancin is more effective than vancomycin	Unclear	Zeng et al., 2016

### Vancomycin

Vancomycin has long been considered the best drug for the treatment of severe MRSA infection, including both HA-MRSA and CA-MRSA which can cause serious, invasive infections such as pneumonia and sepsis (Holmes et al., 2015). Vancomycin has been known as the last line of defense line of against gram-positive cocci infection (Micek, 2007). It was found that the resistance mechanism of vancomycin is mainly specific binding of vancomycinto the bacterial cell wall via peptidoglycan precursor small peptides, which are terminated with D-alanyl-D-alanine; this binding inhibits the elongation and cross-linking of bacterial cell wall peptidoglycans, thereby repressing cell wall synthesis and ultimately leading to bacterial death (Micek, 2007; Haseeb et al., 2019). However, the resistance of *Staphylococcus aureus* to vancomycin is increasing daily, causing widespread concern in the medical community (Haseeb et al., 2019). Currently, a large number of researchers generally divide vancomycin-resistant *Staphylococcus aureus* into three types: vancomycin-resistant *Staphylococcus aureus* (VRSA), vancomycin-intermediate *Staphylococcus aureus* (VISA) and heterologous vancomycin resistant *Staphylococcus aureus* (hetero-VRSA) (Amberpet et al., 2019). VRSA refers to a minimal inhibitory concentration (MIC) of clinically isolated *Staphylococcus aureus* to vancomycin <32 mg/L, and was first reported in the United States in 2002. VISA means that the MIC of *Staphylococcus aureus* to vancomycin is 8–16 mg/L, the first strain was isolated in 1997 in Japan and has attracted the attention of medical community (Baseri et al., 2018). Subsequently, the United States, China and other places have successively discovered multiple VISA (Howden et al., 2010). Hetero-VRSA refers to the primary culture of *Staphylococcus aureus* isolated from clinical specimens. VRSA can be detected by the MH microbroth dilution method or agar dilution method and the MIC of vancomycin is ≤4 mg/L (Severin et al., 2004).

### Daptomycin

Daptomycin is a cyclized lipopeptide drug that is extracted from the fermentation broth of Streptomyces roseosporus (Heidary et al., 2018). Its mechanism of action is to destroy the electric potential of the plasma membranes in the presence of calcium ions, but daptomycin does not inhibit the lipoteichoic acid (Taylor and Palmer, 2016). Due to its unique mechanism of action, daptomycin has no cross-resistance with other antibiotics and can be used to treat skin soft tissue infections and bloodstream infections caused by MRSA, but not MRSA-induced pneumonia because its activity can be suppressed by alveolar surfactant (Gomez Casanova et al., 2017). A large amount of evidence has reported that daptomycin has a faster bactericidal effect than vancomycin, linezolid or quinupristin/dalofopine (Stefani et al., 2015; Gomez Casanova et al., 2017). Furthermore, daptomycin has the effect of resisting most clinical gram-positive bacteria *in vitro;* therefore, daptomycin is mainly applied to treat infections of many drug-resistant bacteria, such as vancomycin-resistant *enterococci*, MRSA, glycopeptide-sensitive *Staphylococcus aureus*, coagulase-negative *Staphylococci*, and penicillin-resistant *Streptococcus pneumoniae* (Chuang et al., 2016). The United States has approved intravenous injections of daptomycin for the treatment of complex skin and soft tissue infections (Heidary et al., 2018). For the dosage form, daptomycin is currently only available in the form of an injection, and its oral dosage form is under study (Mediavilla et al., 2012).

### Linezolid

Linezolid is a synthetic, new class of oxazolidinone antibacterial agents that inhibits *enterococcistaphylococci*, and most strains of *Streptococcus* (Sazdanovic et al., 2016). It is mainly used to control systemic infections caused by vancomycin-resistant *Enterobacter faecium*, such as sepsis, and pneumonia (Krueger and Unertl, 2002). Linezolid can bind to the 23S site of ribosomal RNA on the 50S subunit in bacteria inhibiting the 50S and 30S ribosomal subunits and preventing the formation of the 70S initiation complex, thereby interfering with protein synthesis (Livermore, 2003). This unique mechanism of action eliminates cross-resistance between linezolid and other antibiotics (Livermore, 2003; Hashemian et al., 2018). It has been reported that the survival rate and clinical cure rate of patients with MRSA infection treated with linezolid were significantly higher than those treated with vancomycin. Based on large-scale clinical studies, the oral and injection dosage forms of linezolid are equally effective in the treatment of MRSA, and are also effective against infections such as vancomycin-resistant *enterococci*, penicillin-resistant *pneumococcal*, and macrolide-resistant bacteriostatic *streptococci* (Hashemian et al., 2018). In view of the good therapeutic effect of the drug on multidrug resistant bacteria, it has been used clinically after being approved by the US Food and Drug Administration (FDA) in 2000 (Tyson et al., 2018). In 2007, linezolid also entered the Chinese market. Because it has a strong antibacterial effect on most gram-positive bacteria, it is considered to be an important choice for the treatment of MRSA (Hashemian et al., 2018).

## Novel Therapeutic Strategies for MRSA Treatment

MRSA is multidrug resistant, not only resistant to β-lactam antibiotics, but also resistant to antimicrobial agents such as aminoglycosides, quinolones, and macrolides (Vestergaard et al., 2019). The mortality rate of systemic infection is more than 50%, which has become a worldwide problem in clinical and community anti-infective treatment, and it is difficult to treat (Lindsay, 2013; Vestergaard et al., 2019). At the same time, the hospital ICU is the main site for MRSA, which is likely to cause an outbreak (Lindsay, 2013). Therefore, many new drugs against MRSA are urgently needed. Table 2 lists the newly studied anti-MRSA drugs in recent years.

**Table 2:** Summaries of alternative therapeutic strategies for treatment of MRSA.

**Therapeutic strategies**	**Advantages**	**Disadvantages**	**References**
Quorum sensing inhibition	The quorum sensing inhibitor can block the quorum sensing system of bacteria and inhibit the expression of bacterial virulence genes without affecting the growth and proliferation of bacteria. Therefore, the application of quorum sensing inhibitors can prevent bacteria from developing resistance due to growth stress	The role of quorum sensing inhibitors is relatively narrow and the role of probiotics is unclear	Yin et al., 2011
Lectin inhibition	Lectin inhibition is characterized by high efficiency and low risk, and it is not easy for bacteria to develop drug resistance	The role of lectin inhibition is relatively narrow	Aretz et al., 2018
Iron chelation	Iron carriers play an important role in the control of pathogenic microorganisms. Because of the chelation of iron, the use of iron by pathogenic bacteria can be inhibited, thereby inhibiting the growth and metabolic activity of pathogenic bacteria. Since human cells do not have a related pathway for iron carrier synthesis, their biosynthesis and absorption pathways can also be applied to antimicrobial treatment	The toxicity of iron chelation therapy is relatively large	Borgna-Pignatti and Marsella, 2015
Phage therapy	Phages have many advantages as drugs, such as high specificity, low toxicity, strong reproductive ability, and no cross-resistance with antibiotics	From the discovery of phage to the present, there is no evidence in the past century that phage can cause human diseases, but people still have concerns about their safety. Mainly because it has been reported that phage can mediate the transfer of antibiotic resistance genes and virulence factors, and there are concerns that the host will produce an immune response due to the entry of phage, especially for intravenous administration of phage	Krut and Bekeredjian-Ding, 2018
Nanoparticles	The treatment of nanoparticles has high permeability to bacterial cell membranes and can disrupt the formation of biofilms	The toxicity of iron chelation therapy is relatively large	Li et al., 2010

### Quorum Sensing Inhibition

Quorum sensing is a phenomenon in which bacterial cells regulate the behavior of bacterial populations by sensing self-inducers. Bacteria secrete signal molecules called auto-inducing substances (Perez-Perez et al., 2017). When the extracellular concentration of these substances increases to a certain threshold with the concentration of the population, the bacteria turn on the expression of specific genes, thereby regulating the group behavior of the bacteria (Yin et al., 2011). This is an effective means of information exchange between bacteria, including bioluminescence, biofilm, and toxic gene expression and many other behaviors are regulated by the quorum sensing system (Kalia and Purohit, 2011). The microbial pathogenic properties of *S. aureus* are very complex, and are mainly related to virulence factors (Yin et al., 2011). These virulence factors are chiefly exotoxins that disrupt host cells, interfere with immune responses, and some proteins involved in adhesion and defense against host defenses (Kalia and Purohit, 2011; Yin et al., 2011). The expression of virulence factors is regulated by a complex network composed of multiple genes, of which agr a global regulatory factor H1, is the most important gene regulated by quorum sensing mechanisms (Haseeb et al., 2019). However, the inhibition of this quorum sensing mechanism in bacteria could result in the obstruction of biofilm formation, reduction in bacterial virulence and decreased bacterial resistance (Wang et al., 2017; Haseeb et al., 2019).

### Lectin Inhibition

Lectin is a non-immune-derived sugar-binding protein that enables cell agglutination or precipitation of glycoconjugates (Aretz et al., 2018). It has been reported that lectins can not only agglutinate red blood cells, but also agglutinate with a variety of cells, such as pathogens, immune cells, and germ cells (Aretz et al., 2018; Alghadban et al., 2019). Presently, the application of lectin in the medical field is mainly the specific recognition and adhesion of lectins, which allows various pathogenic microorganisms to bind and infect their recipient cells (Alghadban et al., 2019). For example, certain mannose lectins can significantly affect the toxicity of HIV, which enables the development of antiviral drugs (Barre et al., 2019). Therefore, it is possible to use the characteristics of lectin to design and develop new clinical drugs, and fundamentally prevent the binding of pathogenic microorganisms to recipient cells, thus preventing most infectious diseases (Aretz et al., 2018).

### Iron Chelation

Iron ions are essential nutrients for most organisms, including bacteria (Carver, 2018). Studies have shown that iron ions constitute the catalytic center of important biological enzymes such as oxidoreductase, and participate in various life activities such as electron transport, antioxidant reactions, and nucleic acid synthesis (Nuti et al., 2017). The antibiotic resistance of pathogenic bacteria is continuously increasing; therefore new antibacterial drugs urgently needed (Borgna-Pignatti and Marsella, 2015). One of the important mechanisms of bacterial resistance is to reduce the permeability of the outer membrane and thus hinder the entry of drug molecules into the cells (Carver, 2018). To circumvent the drug resistance mediated by this mechanism, one method is to attach the antibiotic molecule to an iron carrier, forming an iron carrier-antibiotic conjugate, and this iron carrier-antibiotic conjugate can selectively interact with the surface of the bacterial cell membrane (Rayner and Munckhof, 2005). The iron carrier outer membrane receptors interacts with this conjugate; the conjugate then crosses the outer cell membrane by active transport through an iron ion transport system (Bogdan et al., 2016). In this case, the iron carrier bound to the antibiotic can be bound to Fe^3+^, and the resulting complex (antibiotic-iron carrier-Fe^3+^) enters the cell. Finally, the drug is released inside the cell, thereby exerting antibacterial action (Bogdan et al., 2016).

### Phage Therapy

At the beginning of their discovery, phage were used by the former Soviet Union and Eastern European medical communities to treat bacterial infections (Cisek et al., 2017). However, with the introduction of the antibiotic era, people gradually neglected in-depth research on phages. In recent years, due to the increasing global infection rate of drug-resistant bacteria, the use of antibiotics to treat bacterial infections has faced unprecedented challenges (Lin et al., 2017). The emergence of a series of drug-resistant pathogens such as *Staphylococcus aureus, Pseudomonas aeruginosa, Acinetobacter baumannii, tuberculosis, Enterococcus faecalis*, and especially MRSA, has led some scientists and clinicians to refocus their attention on phage research, leading to great progress in this area (Lin et al., 2017; Shlezinger et al., 2017). A large number of experiments have proven that phages can effectively improve the survival rate of animals infected with bacteria (Shlezinger et al., 2017). Compared with antibiotics, phage preparations have the advantages of high specificity, fast self-proliferation, and short development time (Krut and Bekeredjian-Ding, 2018). Phage therapy is considered to be one of the most promising therapies against human pathogens, including antibiotic resistant strains (Shlezinger et al., 2017). As early as 1921, phage were used to treat skin infections caused by *staphylococci* (Wang et al., 2017). In 2007, Italian researchers demonstrated that the phage Msa can effectively control lethal infections caused by *S. aureus* by establishing an intravenous injection model of in mice (Delgado et al., 2000). With the increase of drug-resistant bacteria, the advantages of phages have been recognized by more scholars. However, the biological characteristics of *S. aureus* phages and related animal studies over the years show that there are many limitations in the preparation, storage, and conditions of phages (Lin et al., 2017). Similar to antibiotics, bacteria may also be resistant to phages. However, the diversity and variability of phages in nature also provide an inexhaustible resource pool for phage-controlled bacteria. In addition, phage lysing enzymes that have a destructive effect on the basic framework of bacteria can make up for the lack of phage resistance (Shlezinger et al., 2017). Moreover, at present, phage therapy is still immature in clinical application. The main problems are as follows (Lin et al., 2017; Shlezinger et al., 2017): (1) most phages are highly specific and can only kill one or several subgroups of bacteria; (2) phage therapy in specific *in vitro* test presents effective, but it does not mean that it is equally effective *in vivo*; (3) Phages only begin to proliferate when the bacteria reach a certain density. Phages may be inoculated prematurely or at inappropriate doses, and may be eliminated by the body before they begin to proliferate. Therefore, determining the optimal inoculation time and dose will become a major difficulty in phage treatment. The above are common problems in phage therapy. Therefore, these problems also exist in the course of *Staphylococcus aureus* treatment.

### Nanoparticles

Nanotechnology refers to the preparation, research and industrialization of substances at the nanoscale, as well as comprehensive technical systems for cross-research and industrialization using nanoscale materials (Wu et al., 2020). Studies have shown that nanotechnology can be applied in the fields of medicine, medicine, biology, chemistry, and information technology; therefore, it can play an important role in non-invasive minimally invasive medicine (Barbero et al., 2017). In the medical field, nanoparticles enhance the ability to deliver a drugin the human body (Li et al., 2010). After several layers of nanoparticle-encapsulated smart drugs enter the human body, they can actively search for and attack cancer cells or repair damaged tissues (Wang et al., 2020). China has successfully developed a new generation of nanoscale antibacterial drugs. The powdered nanoparticles are only 25 nanometers in diameter and have strong inhibition and killing effects on pathogenic microorganisms such as *Escherichia coli* and *S. aureus* (Li M. et al., 2017). Nanoscale antibacterial drugs have many properties such as spectrum, hydrophilicity and environmental protection, and they does not produce resistance due to the use of natural minerals (Howden et al., 2010).

## Concluding Remark

*Staphylococcus aureus* is a bacterium that is ubiquitous in the environment and is common in the human body on the surface of the skin and in the upper respiratory tract mucosa (Lowy, 1998). Approximately 20% of human population are a long-term carriers of *S. aureus*, and most people do not show clinical symptoms; however *S. aureus* is still an important pathogen of humans (Humphreys, 2012). *S. aureus* can cause infections in hospitals and communities, and has become the leading pathogen in hospitals worldwide (Edwards and Massey, 2011). In the 1840s, penicillin was discovered by the British bacteriologist Fleming and was used in the clinic to control the *S. aureus* infection (Gardete and Tomasz, 2014). Later, various antibacterial drugs continued to emerge. However, this major research has also laid a hidden danger for human society. The widespread use of antibiotics has led to an increasing incidence of bacterial resistance, beginning with the emergence of multidrug resistant strains such as MRSA, which has been regarded as clinically important problem and has also attracted extensive attention from domestic and foreign research experts (Pichereau and Rose, 2010; Klein et al., 2017). Although the mortality rate of MRSA infection has declined in some European countries in recent years, MRSA is still a serious public health challenge worldwide (Rayner and Munckhof, 2005). Due to its characteristics of easy infection, high mortality and multidrug resistance, MRSA has become a stumbling block in clinical treatment (Khoshnood et al., 2019). Hence, how to effectively prevent and control MRSA has become a hot topic in modern research. Over the years, science and technology have progressed, and medicine has continued to develop. Humans have made outstanding achievements in the research of pathogenic factors of MRSA. Presently, vancomycin is likely still the best drug for curing MRSA infection (Micek, 2007). However, the multidrug resistance of MRSA has greatly increased the difficulty of human research (Vestergaard et al., 2019). Further research is needed to continuously study the ability of MRSA to cause infection and the antibiotic resistance pathways of MRSA, and to promote the development of new drugs against MRSA infection. The development of new drugs has given doctors more options to treat MRSA infections, providing greater protection to human health. However, the efficacy and safety of drugs require further clinical research.

## Author Contributions

YW designed the concept. YG and GS drafted the manuscript. MS collected and analyzed the literature. JW edited the manuscript and revised the language.

### Conflict of Interest

The authors declare that the research was conducted in the absence of any commercial or financial relationships that could be construed as a potential conflict of interest.
